# Compression-Efficient Feature Extraction Method for a CMOS Image Sensor

**DOI:** 10.3390/s26030962

**Published:** 2026-02-02

**Authors:** Keiichiro Kuroda, Yu Osuka, Ryoya Iegaki, Ryuichi Ujiie, Hideki Shima, Kota Yoshida, Shunsuke Okura

**Affiliations:** 1 Graduate School of Science and Engineering, Ritsumeikan University, 1-1-1 Nojihigashi, Kusatsu-shi 525-8577, Japan; ri0113ek@ed.ritsumei.ac.jp (K.K.); ri0088hr@ed.ritsumei.ac.jp (Y.O.); ri0121sp@ed.ritsumei.ac.jp (R.I.); y0sh1d4@fc.ritsumei.ac.jp (K.Y.); 2 Nisshinbo Micro Devices Inc., 3-10 Nihonbashi Yokoyama-cho, Chuo-ku, Tokyo 103-8456, Japan; ujiie.ryuichi@nisshinbo.co.jp (R.U.); shima.hideki@nisshinbo.co.jp (H.S.)

**Keywords:** CMOS image sensor, feature extraction, object recognition, data reduction, run-length encoding

## Abstract

To address the power constraints of the emerging Internet of Things (IoT) era, we propose a compression-efficient feature extraction method for a CMOS image sensor that can extract binary feature data. This sensor outputs six-channel binary feature data, comprising three channels of binarized luminance signals and three channels of horizontal edge signals, compressed via a run length encoding (RLE) method. This approach significantly reduces data transmission volume while maintaining image recognition accuracy. The simulation results obtained using a YOLOv7-based model designed for edge GPUs demonstrate that our approach achieves a large object recognition accuracy (${\mathrm{AP}}_{L}50$) of 60.7% on the COCO dataset while reducing the data size by 99.2% relative to conventional 8-bit RGB color images. Furthermore, the image classification results using MobileNetV3 tailored for mobile devices on the Visual Wake Words (VWW) dataset show that our approach reduces data size by 99.0% relative to conventional 8-bit RGB color images and achieves an image classification accuracy of 89.4%. These results are superior to the conventional trade-off between recognition accuracy and data size, thereby enabling the realization of low-power image recognition systems.

## 1. Introduction

In the present Internet of Things (IoT) era, big data from a vast number of sensors in the physical space is transmitted to cyberspace, where artificial intelligence (AI) processes it and provides feedback to the physical space [1]. To achieve high accuracy in image recognition, Deep Neural Network (DNN) models [2,3,4,5,6] trained with large-scale datasets [7,8,9,10,11] are widely utilized. However, for sustainability purposes, it is important to reduce the power consumption of image recognition systems composed of sensors and AI. In the field of image sensing, CMOS image sensors suitable for AI applications, known as sensing image sensors, have emerged as a promising solution. Conventional RGB color image sensors generate high-resolution photographic images for viewing. Since these images typically contain redundant data that AI removes during feature extraction, sensing image sensors capable of outputting lightweight feature data suitable for AI tasks offer a promising approach to reducing the power consumption of image recognition systems and the storage requirements for massive imaging data [12,13,14,15]. A log-gradient QVGA image sensor [12] outputs feature data in the form of histograms of oriented gradients (HOGs). However, as its readout circuitry is specifically designed for HOG extraction, it cannot produce conventional photographic images. A convolutional image sensor [13] can provide both photographic images and feature outputs; however, it requires in-pixel capacitors to perform multiply–accumulate (MAC) operations. This design results in larger pixel areas, which can degrade light sensitivity and increase fabrication costs. Event-based vision sensors (EVSs) [14,15] are designed to reduce spatial redundancy and enable high dynamic range imaging with compact feature representations. Nevertheless, they face challenges in capturing stationary objects because small luminance variations (those that fall below or rise above a preset threshold [16,17]) are ignored to avoid redundancy. Furthermore, the in-pixel circuits required to detect temporal variations lead to an increase in pixel size.

**Figure 1:**
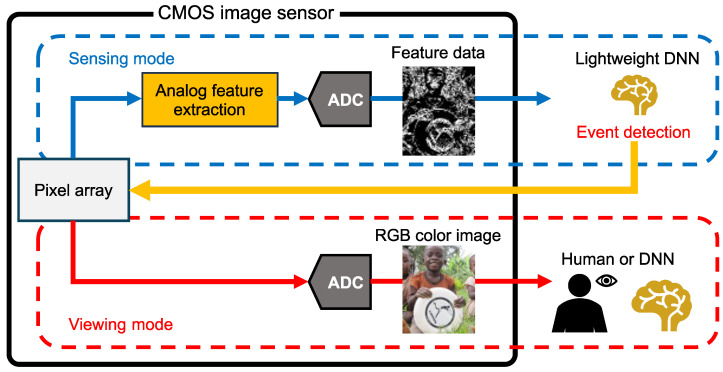
Image recognition system with a feature-extractable CMOS image sensor [8].

Our research group has proposed a CMOS image sensor capable of outputting both photographic RGB color images and lightweight feature data [18,19]. This feature-extractable CMOS image sensor is compatible with conventional RGB color image sensors utilizing small pixels. Figure 1 shows the framework of an image recognition system using the feature-extractable CMOS image sensor with a Bayer pixel array. A key feature of this system is its ability to switch operation modes according to computer vision tasks. In sensing mode, lightweight feature data is processed by a lightweight DNN on the edge device, significantly reducing overall power consumption by eliminating the need to transfer large volumes of image data to cyberspace. With aggressive quantization, the power consumed by ADC is also reduced [18]. When a target object is detected in the feature data as an event, the sensor switches to viewing mode to capture RGB color images for human observation or further analysis by a DNN in cyberspace.

Our research group has also verified an object detection system using binary feature data [20]. First, we developed a lightweight YOLOv7 model based on the YOLOv7 architecture [21], designed to process feature data, specifically horizontal edge information, with low power consumption. This model has approximately the same number of parameters and FLOPs as YOLOv7-tiny, and both models can be implemented on edge-AI devices such as the NVIDIA Jetson Nano. Notably, the object recognition accuracy for large objects (${\mathrm{AP}}_{L}50$) improved by 6.6%. Furthermore, we proposed a feature compression method that combines a feature binarization method and run length encoding (RLE) as an on-chip signal processing technique within a CMOS image sensor. We adopted a feature binarization method based on a given threshold, which was chosen considering recognition accuracy [20]. This threshold-based approach is similar to another work in which the threshold was chosen considering sensor performance and luminance environments [22,23]. The simulation results indicated that, compared with outputting color images, the proposed method reduced the output data size by 99.4%. However, compared with 8-bit edge signals, ${\mathrm{AP}}_{L}50$ decreased by 17.7% because the binary edge signal is insensitive to contrast magnitude (it only indicates whether the absolute gradient is above or below a threshold).

In this paper, we propose a feature data format that combines multiple luminance and edge features, aiming to achieve both high recognition accuracy and high compression efficiency. Specifically, the primary objective is to verify an on-chip signal processing method for the proposed feature data format using large-scale datasets prior to the chip design phase. We also introduce an encoding method using RLE tailored to the feature data characteristics. The details of this on-chip compressed feature extraction method is detailed in Section 2. Section 3 presents the image recognition accuracy and data size, evaluating the proposed CMOS image sensor. Section 5 summarizes this paper.

The main contributions of this study are as follows:

- We present a six-channel binary feature data format consisting of three channels of luminance signals and three channels of horizontal edge signals. This format effectively captures target features across various contrast levels, achieving a superior trade-off between object recognition accuracy and data size compared to previous edge-only methods.
- We propose an effective compression method using run length encoding (RLE) tailored to the six-channel binary feature data characteristics. By allocating an appropriate bit length to the binary values (0 and 1) for each feature channel, this approach significantly reduces data transmission volume while maintaining high accuracy for image recognition tasks.

## 2. Proposed Feature Data Format

In this section, we propose an on-chip signal processing method for a six-channel binary feature data format. Furthermore, we introduce a compression method using RLE tailored to the six-channel binary feature data characteristics.

### 2.1. Overview of Proposed Image Recognition System

**Figure 2:**
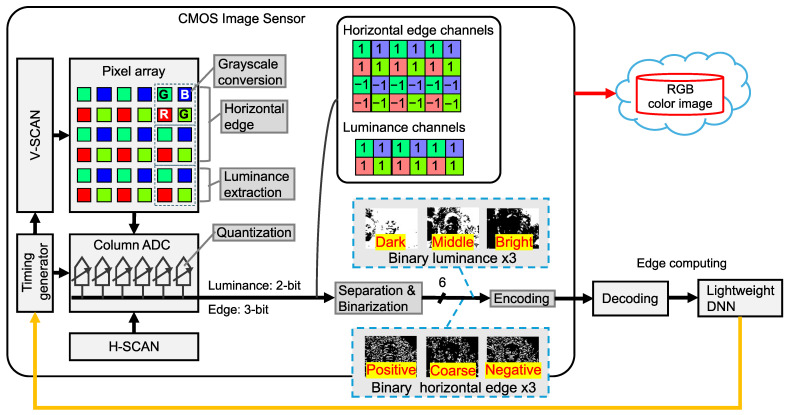
Proposed feature data extraction on CMOS image sensor [8].

Figure 2 shows a more detailed depiction of the system illustrated in Figure 1. The RGB color image is read out using a pixel-by-pixel architecture at an 8-bit resolution, similar to conventional RGB color image sensors. Feature extraction proceeds as follows: First, using its four-shared pixel structure, the sensor bins the RGGB Bayer pixel signal to convert it into a grayscale signal using the formula R+2G+B [24]. This simultaneous readout of photoelectrons from the Bayer array reduces the spatial resolution to one-fourth. Next, instead of performing correlated double-sampling on a single pixel, the horizontal edge is extracted by subtracting the signals from vertically adjacent Bayer cells. This subtraction is realized using a five-transistor (5T) pixel architecture [24,25], which incorporates a vertical binning transistor into the conventional four-transistor (4T) pixel structure. Besides the edge signal, the luminance signal is extracted from Bayer cells adjacent to the horizontal edge extraction cells. Horizontal edge extraction and luminance extraction reduce the spatial resolution to two-thirds, outputting two-channel analog feature data. The analog feature data, consisting of the luminance and horizontal edge signals, is quantized to 2-bit and 3-bit, respectively. We focus on horizontal edges rather than vertical edges because horizontal edge features can be extracted within the pixel array of our proposed CMOS image sensor [26]. Subsequently, the 2-bit luminance signal is separated into three channels of 1-bit luminance signals, representing the dark, middle, and bright regions, respectively. Similarly, the 3-bit horizontal edge signal is separated into three channels of 1-bit horizontal edge signals, representing the fine positive gradients, coarse gradients, and fine negative gradients, respectively. This separation reduces bit transitions within each channel, thereby improving compression efficiency. Quantization, separation, and binarization reduce the total bit resolution to six-eighths compared to the 8-bit RGB color image. Finally, the six-channel binary feature data is compressed using lossless RLE. These processing steps reduce the output data size from the sensor, thereby lowering power consumption [18]. The encoded binary feature data is then decoded and processed using the lightweight DNN model. The proposed on-chip signal compression method of the CMOS image sensor, including six-channel binary feature extraction and tailored RLE, is described in the following sub-sections.

### 2.2. Six-Channel Binary Feature Separation and Binarization

**Figure 3:**
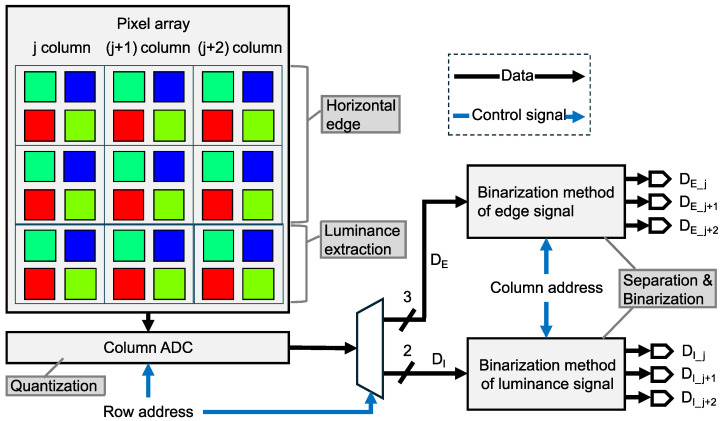
Pipeline of the division and binarization process.

Figure 3 shows the pipeline of the proposed six-channel binary feature separation and binarization. The analog feature data of the horizontal edge and luminance signals extracted from the pixel array are converted into the six-channel binary feature data through quantization by ADCs, followed by separation and binarization using logical operations. Specifically, the analog feature data are quantized by column-parallel ADCs in a row-by-row manner. The horizontal edge signal is quantized to 3 bits and the luminance signal to 2 bits, as described in Section 2.2.1. Subsequently, logical operations described in Section 2.2.2 are sequentially applied to the horizontal edge and luminance signals. For the three columns of 2-bit luminance signals, the j-th, (j+1)-th, and (j+2)-th columns are converted into binary luminance signals representing the dark regions $D_{I\_j}$, the middle regions $D_{I\_j+1}$, and the bright regions $D_{I\_j+2}$, respectively. Similarly, the three columns of horizontal edge signals are converted into binary horizontal edge signals representing the fine positive gradients $D_{E\_j}$, the coarse gradients $D_{E\_j+1}$, and the fine negative gradients $D_{E\_j+2}$, respectively. Consequently, assuming a 1920×1440 Bayer pixel array, each of the resulting feature channels consists of 320×240 pixels.

#### 2.2.1. Quantization for Feature Data

**Figure 4:**
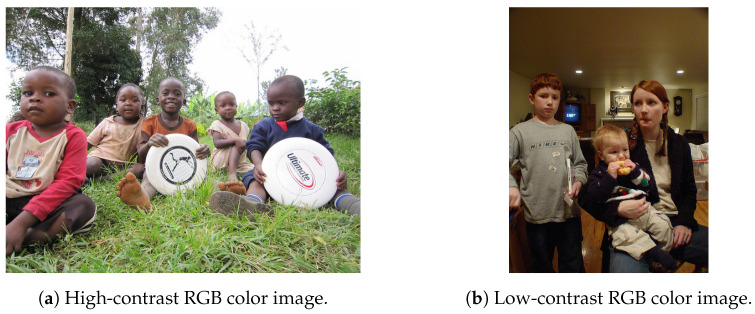
RGB color images [8].

**Figure 5:**
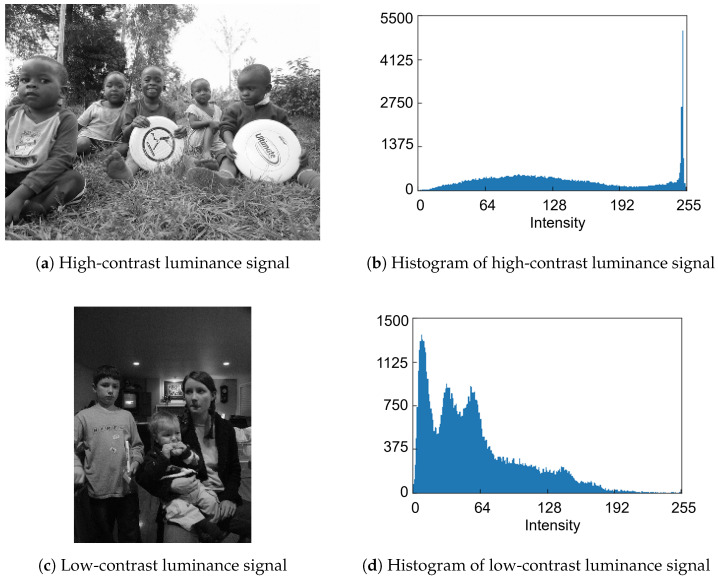
Eight-bit luminance signals and histograms [8].

**Figure 6:**
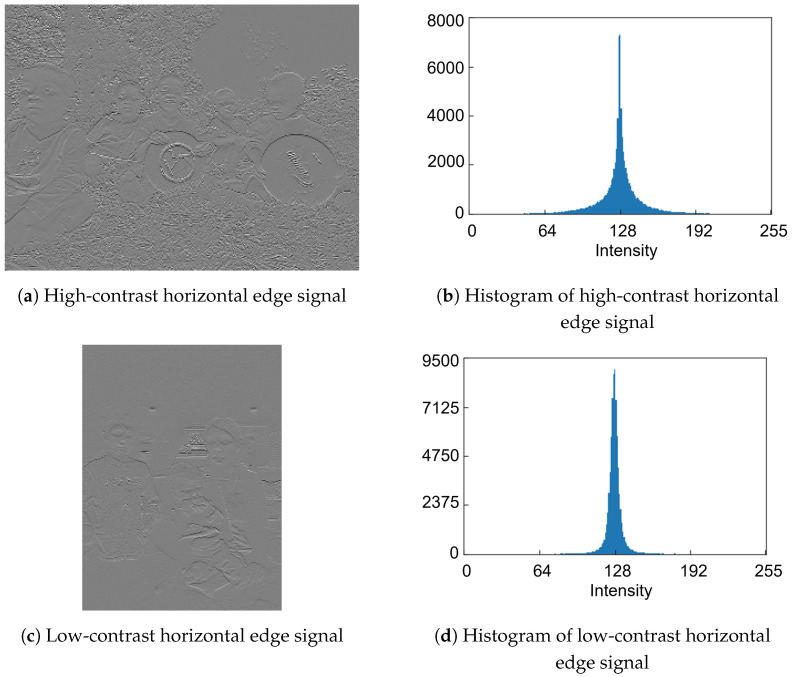
Eight-bit horizontal edge signals and histograms [8].

To decide the quantization strategy for the features, we examine the histogram distributions of feature data generated from RGB color images. Figure 4a,b shows high-contrast and low-contrast RGB color image samples from the COCO dataset, respectively. The histogram distributions of the simulated luminance signals in Figure 5a,c, which are derived from Figure 4a,b, respectively, are shown in Figure 5b,d. Similarly, the histogram distributions of the simulated horizontal edge signals in Figure 6a,c, derived from Figure 4a,b, respectively, are shown in Figure 6b,d. Regarding the luminance signals, the shape of pixel intensity distribution depends on the image scene. In contrast, pixel intensities of the horizontal edge signals exhibit a narrow, centered distribution that is largely independent of the scene, mainly due to the correlation between vertically adjacent Bayer cells. Therefore, while the quantized luminance signal represents the global scene characteristics, it tends to lose local detailed information. Conversely, the quantized horizontal edge signal preserves these local details, thereby complementing the quantized luminance signal. Consequently, both the luminance and horizontal edge signals are expected to be aggressively quantized while maintaining global and local features of the image scene.

**Figure 7:**
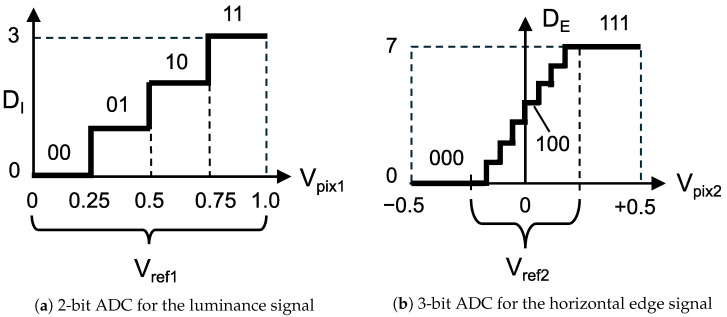
Overview of quantization in a feature-extractable CMOS image sensor.

Figure 7 shows the ADC-based quantization process for analog feature data, based on the histogram characteristics of the luminance and horizontal edge signals. As shown in Figure 7a, the analog luminance signal $V_{\mathrm{pix}1}$ is converted into a 2-bit digital signal $D_{I}$ with a reference voltage range of $V_{\mathrm{ref}1}=1.0\;V$, resulting in a quantization step of 250mV. Regarding the analog horizontal edge signal, since the distribution is concentrated around the center, the ADC reference voltage $V_{\mathrm{ref}2}$ is set to a narrow range centered at 0 V, as shown in Figure 7b. Consequently, the analog edge signal $V_{\mathrm{pix}2}$ is converted into a 3-bit digital signal $D_{E}$. For instance, when the reference voltage $V_{\mathrm{ref}2}$ is set to 250mV, two pixels with $V_{\mathrm{pix}}=0.51\;V$ and $V_{\mathrm{pix}}=0.61\;V$ are quantized into the same luminance value $D_{I}=10$. However, the voltage difference $\Delta V_{\mathrm{pix}}=+0.1\;V$ is captured by the edge signal $D_{E}=101$, representing a positive gradient. In this strategy, the edge signal resolves fine luminance variations that are indistinguishable using the luminance signal alone.

In this paper, we evaluate cases where the reference voltage $V_{\mathrm{ref}2}$ for edge-signal quantization is set to 125mV and 250mV.

#### 2.2.2. Separation and Binarization for Feature Data

Table 1 shows the truth of logical operations used for the separation and binarization of the luminance signal. In this process, $D_{I\_j}$, $D_{I\_j+1}$, and $D_{I\_j+2}$ represent binary luminance features that extract the dark, middle, and bright regions, respectively. Since the 2-bit digital luminance signal $D_{I}$ is divided into 3 channels of 1-bit luminance signals, the data size temporarily increases. However, because bit transitions occur infrequently for gradual luminance variations in the image, the data volume is expected to decrease after compression. Similarly, Table 2 shows the truth of logical operations used for the separation and binarization of the horizontal edge signal. The analog horizontal edge signal intensities above 0V denote a positive gradient, those below 0V indicate a negative gradient, and an intensity of exactly 0V represents no gradient. As the absolute value of the analog edge signal increases, random noise becomes more negligible; however, edges in low-contrast regions are lost. Considering these characteristics of the analog edge signal, $D_{E\_j}$, $D_{E\_j+1}$, and $D_{E\_j+2}$ correspond to the fine positive gradients, coarse gradients, and fine negative gradients, respectively. Since the central codes of the 3-bit horizontal edge signal, specifically “011” and “100”, typically contain random noise, we convert these values to “0”, denoting no gradient. This process suppresses frequent bit transitions caused by noise, thereby improving compression efficiency. However, the magnitude of detectable edges varies depending on the value of $V_{\mathrm{ref}2}$ used for edge-signal quantization. For example, when $V_{\mathrm{ref}2}$ is small, small edges are more likely to be detected, whereas when $V_{\mathrm{ref}2}$ is large, only coarse edges tend to be detected. Therefore, we evaluate two types of logical operations, named types 1 and 2, for the coarse gradients $D_{E\_j+1}$. The logical operation that converts the input codes “001” and “110” to 0 is defined as the “type 1” logical operation, while the logical operation that converts them to 1 is defined as the “type 2” logical operation.

### 2.3. Run Length Encoding (RLE) for Feature Data

RLE compresses binary feature data by counting successive occurrences of 0s and 1s. In this paper, since the binary feature data consists solely of 0s and 1s, we adopt an encoding method that represents the run lengths of 0s and 1s using fixed-bit counters [20]. For instance, given the sentence “0000,0111”, the five consecutive 0s are encoded as “101” and the three consecutive 1s as “011”, assuming 3 bits are allocated to both 0s and 1s. Overflows occur when the number of consecutive 0s or 1s exceeds the capacity of the counter bits. For example, with a 3-bit counter, the sequence “0000,0000” is encoded as “111,000,001”, where the eight 0s are split into two segments (seven 0s and one 0s) to accommodate the overflow. Therefore, achieving high compression efficiency requires selecting a fixed bit width that is well-suited to the data characteristics. To compress the six channels of binary feature data efficiently, we apply the RLE method to each channel individually, selecting an appropriate fixed bit width for each.

**Figure 8:**
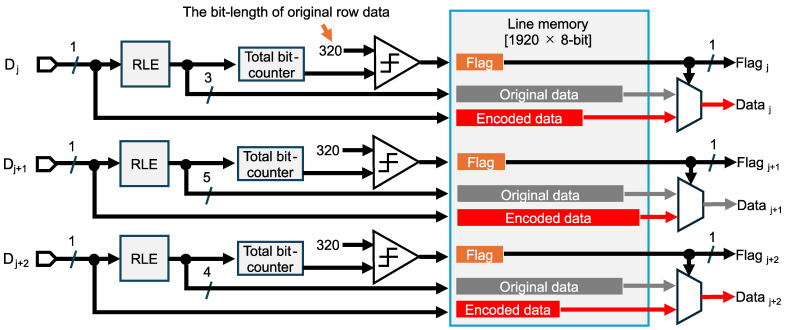
Data flow diagram of run length encoding for the binary feature data to avoid expansion. Separated feature data in the j-th, (j+1)-th, and (j+2)-th columns are denoted as $D_{j}$, $D_{j+1}$, and $D_{j+2}$, respectively.

Figure 8 shows the data flow diagram of RLE for the six-channel binary feature data to avoid expansion. The 2 Mpixel CMOS image sensor typically employs a 1920×8-bit line memory for RGB color images. In the sensing mode, one row of feature data is divided into three channels of binary feature data through the logical operations. Each channel, consisting of 320×1-bit original data, is written to the line memory and then encoded using the proposed run length scheme. Note that one frame of one-channel feature data consists of 320×240 pixels, supposing a 1920×1440 Bayer pixel array. Since the characteristics of feature data differ by channel, we employ separate RLE compressors with distinct fixed-bit counters for each channel to improve the compression efficiency. For example, when a 3-bit length is allocated to the counters for 0s and 1s, the consecutive lengths of 0s and 1s are alternately written into the available space in the line memory. The encoded total bit length is simultaneously counted for each channel. If the counted length is less than 320, the compression rate is less than 100% and the encoded data is output along with a flag indicating successful compression. Conversely, if the compression rate exceeds 100%, that means data volume expansion is caused by frequent 0/1 transitions and overflows, and the original 320×1-bit data stored in the line memory is output without encoding. This selection process is performed independently for each channel to keep the compression rate below 100%.

## 3. Simulation Results

**Figure 9:**
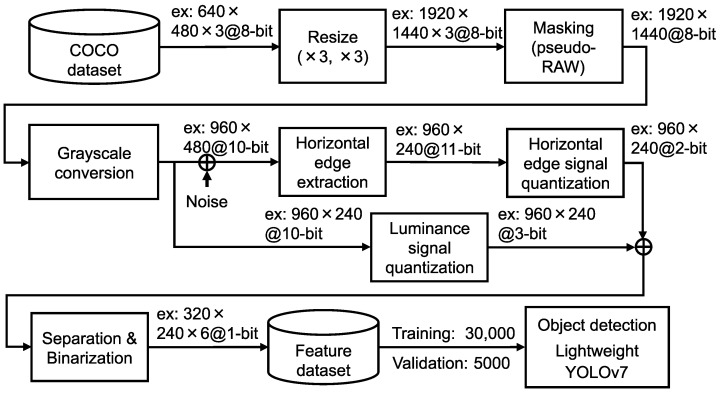
Processing flow to generate six channels of binary feature data.

To evaluate object recognition accuracy with the proposed CMOS image sensor, we converted RGB color images from the COCO dataset [8] into the feature dataset, as shown in Figure 9. Since the majority of images in the COCO dataset have a resolution of 640×480 pixels, the data were first resized to approximately 2 Mpixels to match the resolution required by the proposed CMOS image sensor. Furthermore, the three-channel RGB color image was masked to emulate the pixel output of a one-channel raw image, where each pixel value follows the Bayer pattern. The luminance signal was generated through grayscale conversion, and the horizontal edge signal was obtained by adding estimated pixel reset noise. This noise addition emulates the uncancelled Floating Diffusion (FD) reset noise caused by the dual reset pulsing scheme [19]. Subsequently, quantization, separation, and binarization processes were applied to the luminance and horizontal edge signals, resulting in the six-channel binary feature data. The feature dataset consists of 30,000 training images and 5000 validation images. To evaluate object recognition accuracy, we used the lightweight YOLOv7 model [20], with its input layer modified to accept six channels. The detailed model configuration, which is shown in Table 3, follows the architecture described in our previous work [20]. The model was trained from scratch without using pre-trained weights. The training hyperparameters are shown in Table A1.

### 3.1. Six-Channel Binary Feature Data

**Figure 10:**
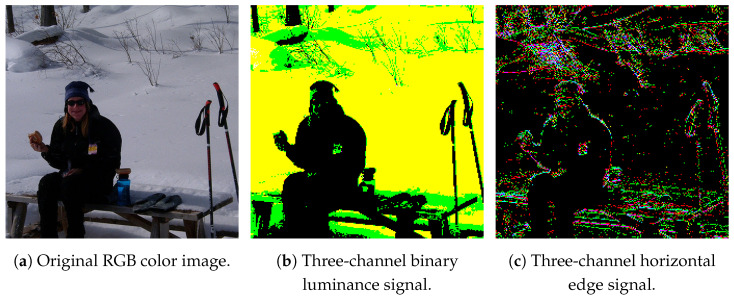
Sample six-channel binary feature data [8].

Figure 10 displays sample six-channel binary feature data. Figure 10a presents the original RGB color image. The simulated three-channel luminance signal and horizontal edge signal are visualized as three-channel images, as shown in Figure 10b,c, respectively. Since the luminance signals primarily capture global signal intensity, noise on the snow caused by fine gradients is largely absent in the luminance signal but appears in the horizontal edge signal. Conversely, the horizontal edge signal extracts local detailed information, rendering the outlines of the glasses, mouth, and head, which are indistinct in the luminance signal, clearly visible. Consequently, we expect that objects can be detected by utilizing the luminance signal as the primary feature for global scene characteristics and the horizontal edge signal for local details.

Table 4 summarizes the impact of the reference voltage $V_{\mathrm{ref}2}$ used for edge-signal quantization and of the logical operations used for separation and binarization on object recognition accuracy. Object recognition accuracy is quantified using AP50 (mean average precision at an IoU of 0.50) and ${\mathrm{AP}}_{L}50$ (accuracy for large objects only). We focus on large objects because the proposed system assumes that if a given object is detected in the feature data, further analysis, such as detecting small objects, will be performed using the RGB color images. Comparing object detection accuracy across different logical operations reveals that when $V_{\mathrm{ref}2}=125\;\mathrm{mV}$, the type 1 logical operation achieved a higher ${\mathrm{AP}}_{L}50$. In contrast, when $V_{\mathrm{ref}2}=250\;\mathrm{mV}$, the type 2 logical operation yielded higher performance for both AP50 and ${\mathrm{AP}}_{L}50$. Therefore, in the following evaluations, we adopt the type 1 logical operation for $V_{\mathrm{ref}2}=125\;\mathrm{mV}$ and the type 2 logical operation for $V_{\mathrm{ref}2}=250\;\mathrm{mV}$.

### 3.2. Compression Rate of Tailored RLE

**Figure 11:**
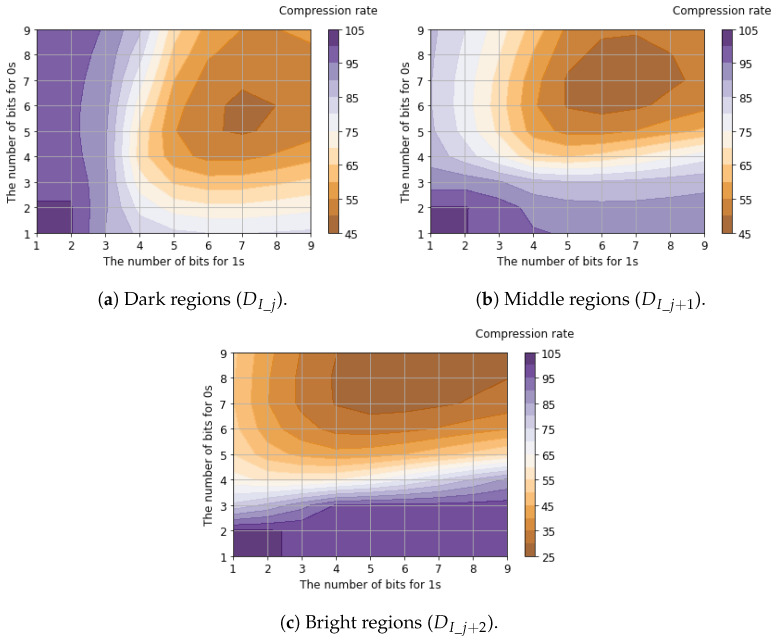
Compression rates of luminance signals with respect to bit allocation.

**Figure 12:**
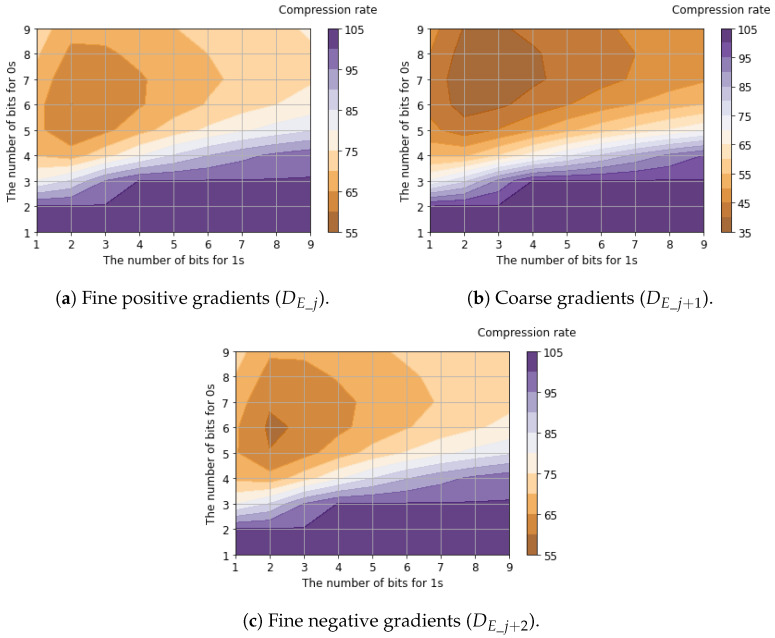
Compression rates of horizontal edge signals ($V_{\mathrm{ref}2}=125\;\mathrm{mV}$) with respect to bit allocation.

**Figure 13:**
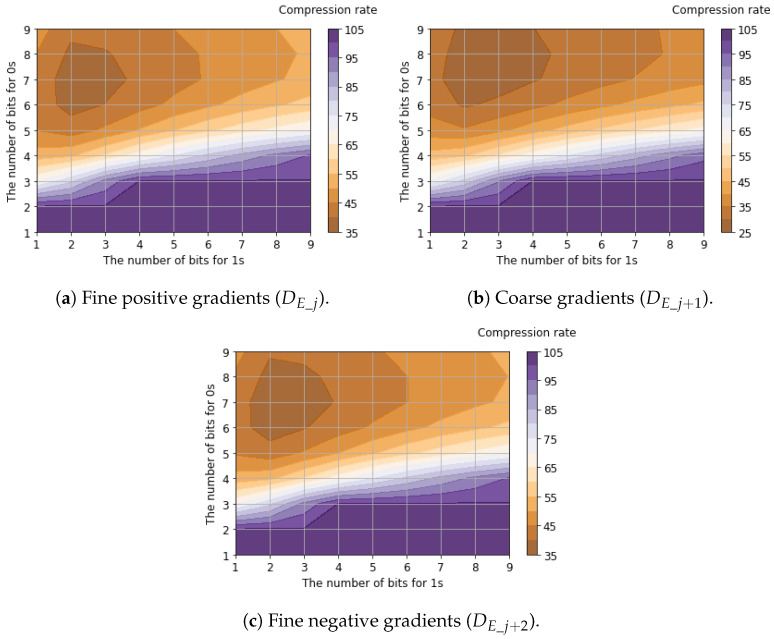
Compression rates of horizontal edge signals ($V_{\mathrm{ref}2}=250\;\mathrm{mV}$) with respect to bit allocation.

Table 5 shows the compression rates for bit allocation applying the proposed tailored RLE method to the six-channel binary feature data. The table presents the best compression rate among all possible bit allocation patterns, evaluated using 25,000 samples of the six-channel binary feature data. Detailed compression rates for each channel with respect to bit allocation are shown in Figure 11, Figure 12 and Figure 13.

Regarding luminance signals, we found that allocating balanced fixed-bit counters for 0s and 1s yields the minimum compression rates. This is because luminance signals capture the global signal intensity where bit transitions are suppressed due to the spatial correlation between horizontally adjacent pixels. In contrast, the compression rates of horizontal edge signals are minimized when allocating a larger bit width for 0s (denoting the absence of an edge gradient) compared to 1s (denoting the presence of an edge gradient). This occurs because horizontal edge signals capture local intensity changes. Since these changes appear sparsely, and the run length of 1s is significantly shorter than that of 0s. Compared to the case where $V_{\mathrm{ref}2}=125\;\mathrm{mV}$, improved compression efficiency (a lower compression ratio) was achieved when $V_{\mathrm{ref}2}=250\;\mathrm{mV}$. This improvement occurred because increasing $V_{\mathrm{ref}2}$ reduces sensitivity to small edges, thereby increasing the proportion of consecutive 0s that represent the absence of a gradient. As a result, the average compression rates for all six channels of binary feature data were calculated to be 46.5% for $V_{\mathrm{ref}2}=125\;\mathrm{mV}$ and 37.5% for $V_{\mathrm{ref}2}=250\;\mathrm{mV}$.

### 3.3. Data Size and Object Detection Accuracy

**Figure 14:**
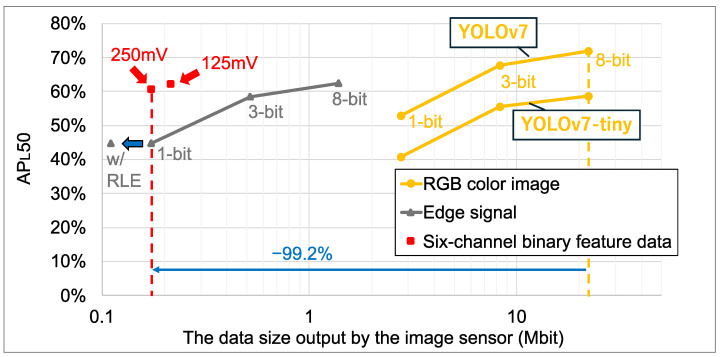
Data size and object recognition accuracy.

Figure 14 shows the object recognition accuracy (${\mathrm{AP}}_{L}50$) and data sizes for our lightweight YOLOv7 model trained on feature data [20]. For reference, the accuracy of the YOLOv7 and YOLOv7-tiny models trained on RGB color images is also plotted. The feature data includes six-channel binary feature data generated by the proposed method and edge-only signals from our previous works [19,20]. The input layer resolution of all models was matched to the resolution of the proposed sensor output feature data. For the six-channel binary feature data, the plotted data size reflects the size after compression using the tailored RLE method. The results indicate that the six-channel binary feature data achieved a superior trade-off between recognition accuracy and data size. Detailed numerical results are provided in Table 6, Table 7 and Table 8. The COCO dataset with 80 object categories is utilized for validation. Compared to the 3-bit edge signal, the size of the six-channel binary feature data with $V_{\mathrm{ref}2}=125\;\mathrm{mV}$ was reduced by 58.7%, and object recognition accuracy was improved by 3.7% (62.1−58.4%). Furthermore, compared to conventional 8-bit RGB color images and YOLOv7-tiny, the feature data with $V_{\mathrm{ref}2}=250\;\mathrm{mV}$ improved object recognition accuracy by 2.0% (60.7−58.7%) while reducing the data size by 99.2%. Notably, our lightweight YOLOv7 model trained on six-channel features has approximately the same number of parameters as YOLOv7-tiny, with a reduction of 0.3 M. In addition, the FLOPs are reduced by 8.3 G (13.7−5.4G) compared to YOLOv7-tiny, primarily due to the difference in the spatial resolution between the RGB color image and feature data.

### 3.4. Evaluation on Image Classification

To examine the robustness of the proposed feature data format for different image recognition tasks in the sensing mode, we converted RGB color images from the Visual Wake Words (VWW) dataset [11] into the six-channel binary feature dataset, assuming a binary classification task to detect the presence of a person. Since the VWW dataset filters labels into “person” and “not-person” categories based on the COCO dataset, six-channel binary feature data based on the VWW dataset were generated using the same method shown in Figure 9. The images containing a “person” are labeled as 1 only when the bounding box is “Large” and “Medium” according to the COCO dataset definition. To evaluate image classification accuracy, we used the MobileNetV3-Large 1.0 model [27] tailored for mobile devices, with its input layer modified to accept six channels. The model was trained from scratch without using pretrained weights. For evaluation, in addition to the standard VWW validation set, we employed the Wake Vision (WV) dataset [28]. While the VWW dataset is widely used, it contains label errors that can affect evaluation reliability. The WV dataset cleans these labels, providing a more rigorous benchmark [28].

**Figure 15:**
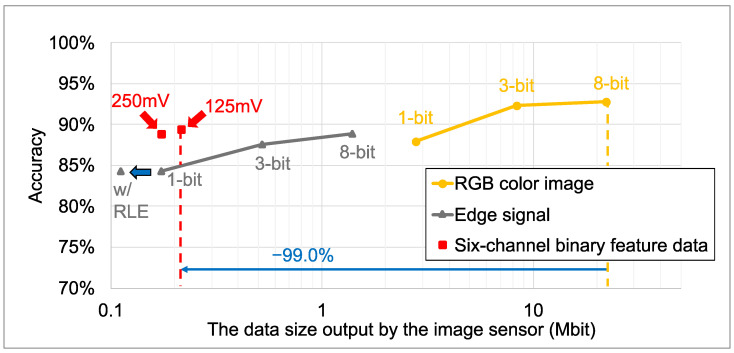
Data size and image classification accuracy.

Figure 15 shows the image classification accuracy and data size for the MobileNetV3 model trained on quantized RGB color images and feature data generated from the VWW dataset. The feature data include six-channel binary feature data generated by the proposed method and edge-only signals from our previous works. Since image classification typically requires lower resolution than object detection, all image data were resized to 256×256 pixels instead of using the raw sensor output resolution. For the six-channel binary feature data and the previous 1-bit edge signal, the plotted data size reflects the size after compression using the tailored RLE method. The training process and learning curves for each data format are detailed in Appendix B. Detailed numerical results are provided in Table 9 and Table 10. The simulation results show that the six-channel binary feature data yielded superior image classification accuracy, similar to the object detection task using the YOLOv7 models and the COCO dataset. Compared to the 8-bit edge signal, six-channel binary feature data with $V_{\mathrm{ref}2}=125\;\mathrm{mV}$ reduced the feature data size by 84.5%, and image classification accuracy improved by 0.5%. Furthermore, compared to conventional 8-bit RGB color images, the six-channel binary feature data reduced the data size by 99.0% at the cost of a 3.4% accuracy degradation. Evaluation on the WV dataset showed similar trends, confirming the robustness of the proposed features against label noise.

## 4. Discussion

For both the large object detection and binary person classification tasks assumed in sensing mode, the proposed method demonstrated a superior trade-off between recognition accuracy and data size, compared to the RGB color image. These results demonstrate that our image recognition system can detect target objects in an energy-efficient manner; these objects serve as triggers to switch the operation mode of the CMOS image sensor from the sensing mode to the viewing mode, thereby realizing a low-power image recognition system.

Table 11 compares the proposed method with other feature extraction sensors. First, our method does not utilize in-pixel capacitors, making it well suited for low-cost CMOS image sensors with small pixels. Second, our sensor can output both RGB color images and feature data. This capability enables a flexible image recognition system configuration. Finally, the proposed method has been validated using large-scale datasets. While other methods are often validated on limited scenarios such as face or hand detection, our method has been verified using standard benchmarks (COCO, VWW, and WV), demonstrating its applicability to realistic scenarios.

## 5. Conclusions and Future Work

To realize an energy-efficient image recognition system, we proposed a compression-efficient feature extraction method for a CMOS image sensor capable of extracting binary feature data and present simulation results regarding recognition accuracy and compression efficiency. The compressed feature data is extracted via six-channel feature extraction and encoded using run length encoding with an appropriate bit length to binary values (0 and 1) for each feature channel. The simulation results obtained using our lightweight YOLOv7 model demonstrate that our approach improved the ${\mathrm{AP}}_{L}50$ by 2.0% on the COCO dataset and reduced the data size by 99.2%, relative to conventional 8-bit RGB color images and the YOLOv7-tiny model. Furthermore, the image classification results obtained using MobileNetV3 show that our approach reduced the data size by 99.0% at the cost of a 3.4% accuracy degradation relative to conventional 8-bit RGB color images. These results indicate that the proposed method offers a superior trade-off between recognition accuracy and data size, thereby realizing a low-power image recognition system.

In future work, we plan to carry out hardware implementation to validate the system’s practical performance. Building on our previous chip-level studies on pixels and ADCs [24,26], we aim to fabricate a prototype chip to validate the system’s practical performance and power efficiency in real-world environments.

## Figures and Tables

**Table 1:** The logical operations used for the division and binarization of the luminance signal.

$D_{I}$<1:0>	$D_{I\_j}$	$D_{I\_j+1}$	$D_{I\_j+2}$
00	0	0	0
01	1	0	0
10	1	1	0
11	1	1	1

**Table 2:** The logical operations used for the division and binarization of the horizontal edge signal.

$D_{E}$<2:0>	$D_{E\_j}$	$D_{E\_j+1}$		$D_{E\_j+2}$
		Type 1	Type 2	
000	0	1	1	1
001	0	0	1	1
010	0	0	0	1
011	0	0	0	0
100	0	0	0	0
101	1	0	0	0
110	1	0	1	0
111	1	1	1	0

**Table 3:** Comparison of model configurations.

Model	Channel Width	Backbone ELAN Depth	Neck ELAN Depth	#Param.
YOLOv7	100%	6	6	36.9 M
Ours	50%	6	3	5.9 M
YOLOv7-tiny	50%	4	4	6.2 M

**Table 4:** Object recognition accuracy after six-channel feature extraction.

*V*_ref2_	Logical Operation	AP50	AP_L_50
125 mV	type 1	36.6%	62.1%
	type 2	36.6%	61.7%
250 mV	type 1	34.5%	59.1%
	type 2	35.6%	60.7%

**Table 5:** Compression rates and fixed-bit allocation obtained using the proposed lossless RLE method for the feature data after division and binarization.

Data		#Allocating Bits		Comp. Rate	
		0s Runs	1s runs	Each Channel	Average
Luminance signals	$D_{I\_j}$	6-bit	7-bit	49.1%	41.1%
	$D_{I\_j+1}$	7-bit	6-bit	47.4%	
	$D_{I\_j+2}$	8-bit	6-bit	26.8%	
Edge signals ($V_{ref2}=125$)	$D_{E\_j}$	6-bit	2-bit	59.9%	51.9%
	$D_{E\_j+1}$	7-bit	2-bit	36.6%	
	$D_{E\_j+2}$	6-bit	2-bit	59.1%	
Edge signals ($V_{ref2}=250$)	$D_{E\_j}$	7-bit	2-bit	37.5%	33.8%
	$D_{E\_j+1}$	7-bit	2-bit	27.0%	
	$D_{E\_j+2}$	7-bit	2-bit	36.9%	

**Table 6:** Object recognition accuracy and data size in quantized RGB color images and YOLOv7-tiny.

Bit Depth	8-Bit	3-Bit	1-Bit
Data size (bits)	22,118,400	8,294,400	2,764,800
AP50	48.1%	45.3%	31.6%
${\mathrm{AP}}_{L}50$	58.7%	55.5%	40.7%

YOLOv7-tiny: #Param. = 6.2 M, FLOPs = 13.7 G; the resolution of the model’s input layer is 640×640.

**Table 7:** Object recognition accuracy and data size in quantized horizontal edge feature data.

Bit Depth	8-Bit	3-Bit	1-Bit	1-Bit + Comp.
Data size (bits)	1,382,400	518,400	172,800	110,765
AP50	44.9%	39.8%	27.6%	
${\mathrm{AP}}_{L}50$	62.4%	58.4%	44.7%	

Lightweight YOLOv7 [20]: #Param. = 5.9 M, FLOPs = 11.8 G; the resolution of the model’s input layer is 480×480 to meet the CMOS image sensor [20].

**Table 8:** Object recognition accuracy and data size in six-channel binary feature data.

*V*_ref2_	125 mV	125 mV + Comp.	250 mV	250 mV + Comp.
Data size (bits)	460,800	214,281	460,800	172,595
AP50	36.6%		35.6%	
${\mathrm{AP}}_{L}50$	62.1%		60.7%	

Lightweight YOLOv7 for six channels of features: #Param. = 5.9 M, FLOPs = 5.4 G; the resolution of the model’s input layer is 320×320 to meet the proposed CMOS image sensor.

**Table 9:** Image classification accuracy and data size in quantized RGB color images.

Bit Depth	8-Bit	3-Bit	1-Bit
Data size (bits)	22,118,400	8,294,400	2,764,800
Accuracy (VWW)	92.8%	92.3%	87.9%
Accuracy (WV)	86.3%	85.5%	80.8%

**Table 10:** Image classification accuracy and data size in feature data.

Feature Type	Edge Signal			Six-Channel Binary Feature Data	
Data format	8-bit [19]	3-bit [19]	1-bit [20]	$V_{\mathrm{ref}2}=125\;\mathrm{mV}$	$V_{\mathrm{ref}2}=250\;\mathrm{mV}$
Data size (bits)	1,382,400	518,400	110,765	214,281	172,595
Accuracy (VWW)	88.9%	87.6%	84.3%	89.4%	88.9%
Accuracy (WV)	81.0%	80.8%	76.1%	81.3%	80.6%

**Table 11:** Comparison of the proposed method with other feature extraction sensors.

	This Work	[12]	[13]	[22]	[29]
**Feature extraction**	Luminance and Edge (Analog)	HOG (Analog)	3×3 Conv. (Analog)	Windmill Edge (Analog)	3×3 Conv. (Digital)
**Pixel structure**	5T	4T	4T1C	4T	8T2C
**RGB color image**	Available	Not available	Available	Available	Available
**Validation data**	COCO/ VWW/WV	PASCAL VOC [9] PASCAL RAW [30]	N/A	Face/Hand	Face

**Figure A1:**
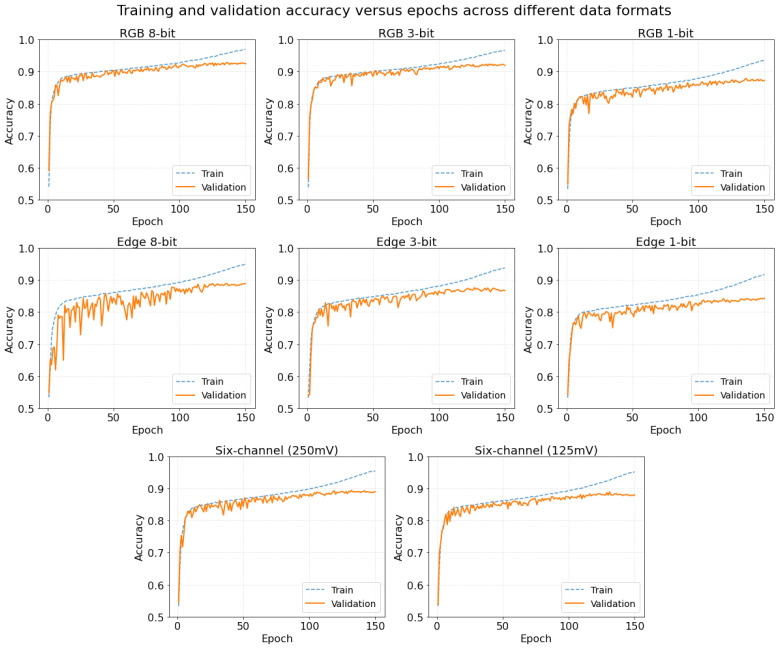
Epochs and accuracy during training on the VWW dataset.

## Data Availability

The original contributions presented in this study are included in this article. Further inquiries can be directed to the corresponding authors.

## Abbreviations

The following abbreviations are used in this manuscript:

CMOS	Complementary Metal–Oxide Semiconductor
RGB	Red, Green, and Blue
QVGA	Quarter Video Graphics Array
ADC	Analog-to-Digital Converter
FLOPs	Floating Point Operations per Second
IoU	Intersection over Union

## Appendix A. Training Hyperparameters

To ensure the reproducibility of our simulation results, the detailed training hyperparameters used for our lightweight YOLOv7 model are listed in Table A1. We strictly followed the settings of the original YOLOv7 model [21]. The model was trained using the SGD optimizer for 400 epochs. We performed a warm-up for 3 epochs, updating only the bias during this stage. Subsequently, the learning rate was linearly decayed from an initial value of 0.01 to a final value of 0.001. The detailed data augmentation settings are also provided in the table.

**Table A1:** Hyperparameter settings for the lightweight YOLOv7 model [20].

Hyperparameter	Value
Epochs	400
Optimizer	SGD
Initial learning rate	0.01
Final learning rate	0.001
Momentum	0.937
Weight decay	0.0005
Warm-up	
Warm-up epochs	3
Warm-up momentum	0.8
Warm-up bias learning rate	0.1
Loss Gains	
Box loss gain	0.05
Class loss gain	0.3
Obj loss gain	0.7
Data Augmentation	
HSV hue	0.015
HSV saturation	0.7
HSV value	0.4
Translation	0.2
Scale	0.9
Horizontal flip	0.5
Mosaic	1.0
MixUp	0.15
Copy & Paste	0.15

Note: SGD denotes Stochastic Gradient Descent. HSV stands for Hue, Saturation, and Value.

## Appendix B. Learning Curves

Figure A1 shows the learning curves (training and validation accuracy versus epochs) when the model is trained using the VWW dataset across different data formats. The learning curves demonstrate that the validation accuracy converges stably alongside the training accuracy. Therefore, we conclude that our models have successfully learned features from the VWW dataset to evaluate our feature extraction method compared to the RGB color image.
